# Clinical Evaluation of Roche SD Biosensor Rapid Antigen Test for SARS-CoV-2 in Municipal Health Service Testing Site, the Netherlands

**DOI:** 10.3201/eid2705.204688

**Published:** 2021-05

**Authors:** Zsὁfia Iglὁi, Jans Velzing, Janko van Beek, David van de Vijver, Georgina Aron, Roel Ensing, Kimberley Benschop, Wanda Han, Timo Boelsums, Marion Koopmans, Corine Geurtsvankessel, Richard Molenkamp

**Affiliations:** Erasmus MC, Rotterdam, the Netherlands (Z. Iglὁi, J. Velzing, J. van Beek, D. van de Vijver, G. Aron, M. Koopmans, C. Geurtsvankessel, R. Molenkamp);; Public Health Service Rotterdam-Rijnmond, Rotterdam (R. Ensing, T. Boelsums);; National Public Health Institute (RIVM), Bilthoven, the Netherlands (K. Benschop, W. Han)

**Keywords:** COVID-19, coronavirus disease, SARS-CoV-2, severe acute respiratory syndrome coronavirus 2, viruses, respiratory infections, zoonoses, Roche, rapid antigen test, clinical evaluation, diagnostics, the Netherlands

## Abstract

Rapid detection of infection is essential for stopping the spread of severe acute respiratory syndrome coronavirus 2 (SARS-CoV-2). The Roche SD Biosensor rapid antigen test for SARS-CoV-2 was evaluated in a nonhospitalized symptomatic population. We rapid-tested a sample onsite and compared results with those from reverse transcription PCR and virus culture. We analyzed date of onset and symptoms using data from a clinical questionnaire. Overall test sensitivity was 84.9% (95% CI 79.1–89.4) and specificity was 99.5% (95% CI 98.7–99.8). Sensitivity increased to 95.8% (95% CI 90.5–98.2) for persons who sought care within 7 days of symptom onset. Test band intensity and time to result correlated strongly with viral load; thus, strong positive results could be read before the recommended time. Approximately 98% of all viable specimens with cycle threshold <30 were detected. Rapid antigen tests can detect symptomatic SARS-CoV-2 infections in the early phase of disease, thereby identifying the most infectious persons.

Severe acute respiratory syndrome coronavirus 2 (SARS-CoV-2) emerged >1 year ago (*1*) but still keeps a strong grip not only on daily life but also on diagnostic capacities. Reverse transcription PCR (RT-PCR) has been the standard for diagnosis of acute infection (*2*) but has several limitations, such as the requirement for specialized laboratory infrastructure, trained personnel, and reagents that have been in shortage globally (*3*). In addition, the current turnaround time from sample collection to reporting of the result may take >48 hours (J. van Beek et al., unpub. data, https://doi.org/10.1101/2020.10.13.20211524), compromising effectiveness of triage, isolation, and contact tracing strategies. Rapid antigen detection tests (Ag RDT) for SARS-CoV-2 appeared on the market in early 2020, but initial reports of poor performance and the lack of independent evaluation results made governments reluctant to invest and consider inclusion into testing algorithms. As of February 2021, more than 140 assays are on the market (*4*), but relatively few have been extensively validated (*4*–*5*; V.M. Corman et al., unpub. data. https://doi.org/10.1101/2020.11.12.20230292). Initial results show that these tests are suitable for detecting early-onset cases with high viral load. As expected, the sensitivity of the tests is lower than that of RT-PCR, but in patients in the early phase of illness who have high viral load, performance meets World Health Organization–set criteria of >80% sensitivity and >97% specificity compared with nucleic acid detection methods (*6*). Thus, these tests could be useful in identifying the most infectious persons (J. van Beek et al.). In an outbreak scenario, diagnostics with lower sensitivity but a faster result can render interventions more effective than standard tests (*7*). Implementation of Ag RDT into testing algorithms would enable rapid detection and isolation of new cases and thereby support the test, trace, and isolate strategy with the intent to stop transmission chains and reduce the impact of coronavirus disease (COVID-19).

In this study, we assessed the performance of the Roche SD Biosensor SARS-CoV-2 rapid antigen test (Roche Diagnostics, https://www.roche.com) compared with both RT-PCR and virus culture. We conducted the field evaluation study at a large public health service testing facility in Rotterdam-Rijnmond, the Netherlands, where most visitors sought care for COVID-19 symptoms. Every person >18 years of age who had an appointment for SARS-CoV-2 RT-PCR testing was invited to participate. An additional nasopharyngeal swab specimen was obtained for the Ag RDT in parallel and processed onsite to compare sensitivity and specificity to RT-PCR. All samples positive by Ag RDT and PCR were cultured to correlate results with infectivity. The medical research ethics committee of Utrecht decided the study was not subject to the Medical Research Involving Human Subjects Act and did not require full review by an accredited committee (protocol no. 20-606/C).

## Materials and Methods

### Testing Population, Setup and Patient Recruitment

The study was conducted at the largest drive-through testing location in Rotterdam-Rijnmond, at which testing is by appointment only. Eligibility for a free-of-charge test included either presence of symptoms or close contact with a confirmed SARS-CoV-2–infected person. Most persons who requested testing had symptoms. At the entrance of the testing site, we approached all persons >18 years of age; after providing written informed consent, they were enrolled in the study and directed to one of the dedicated testing posts for sampling. Enrolled persons were also asked to fill in a clinical questionnaire stating the reason for appointment, date of onset or end date of symptoms, and a list of symptoms (fever, sore throat, coughing, shortness of breath/tightness, runny nose, diarrhea, eye complaints, nausea, rash, chills, headache, pain when breathing, coughing phlegm, muscle pain, painful/swollen lymph nodes, fatigue, vomiting, joint pain, loss of appetite, nosebleed, other). The study was conducted for 5 days to achieve the target of 800–1,000 participants. The SARS-CoV-2 rapid antigen test distributed by Roche SD Biosensor was provided by the Ministry of Health, Welfare, and Sport. 

### Testing Site Setup and the Mobile Laboratory

From the 6 available testing posts, we designated 2 posts for sample collection from study participant on the basis of 3 factors: maximum number of subjects per test post (≈150/day); known number of appointments per day; and expected enrollment rate based on initial results from other study sites in the Netherlands. We expected to include a maximum of 300 persons/day. The site’s regular trained personnel performed swabbing to avoid variations to the process. Testing was done on benchtop, in a mobile laboratory unit by trained staff dressed in full personal protective equipment (goggles, FFP3 mask, gloves, and disposable gown). Samples for the Ag RDT were collected at regular intervals and processed as soon as possible within 30 mins in convenient batches (5–10 tests at a time). Swab specimens and RDT devices were inactivated in chlorine and disposed of as biohazard material. Results were recorded in a Microsoft Access database (https://www.microsoft.com) designated for this study. 

### Specimen Collection, Testing and Culture Procedures

Standard method for SARS-CoV-2 testing was by RT-PCR, which was conducted in parallel with the Ag RDT on separate swab specimens. Two swab specimens (1 oropharyngeal and 1 nasopharyngeal swab) were taken for RT-PCR and virus culture, placed directly in 3 mL universal transport media (HiViral; HiMedia Laboratories PVT, Ltd., https://www.himedialabs.com) and shipped to the Erasmus MC viroscience diagnostic laboratory (Rotterdam, the Netherlands). For the Ag RDT evaluation, a second nasopharyngeal swab specimen was taken from the same nostril, using the swab included in the kits, to directly compare RT-PCR results with Ag RDT results. Swabs were placed into empty tubes to transport to the mobile laboratory onsite. Routine RT-PCR testing was performed on combined oropharyngeal and one nasopharyngeal swabs in virus transport medium using the cobas SARS-CoV-2 test on the COBAS6800 (Roche Diagnostics). Because cycle threshold (C_t_) values differ between PCR methods, genome copies per milliliter were calculated based on an in-house established standard curve. The leftover virus transport medium from the oropharyngeal and nasopharyngeal swabs was directly inoculated onto Vero cells clone 118 without freezing or extended storage. Samples were cultured for 7 days; once cytopathic effect was visible, the presence of SARS-CoV-2 was confirmed with immunofluorescent detection of SARS CoV-2 nucleocapsid protein (rabbit polyclonal antibody; Sino Biologic Inc., https://www.sinobiological.com).

**Figure 1:**
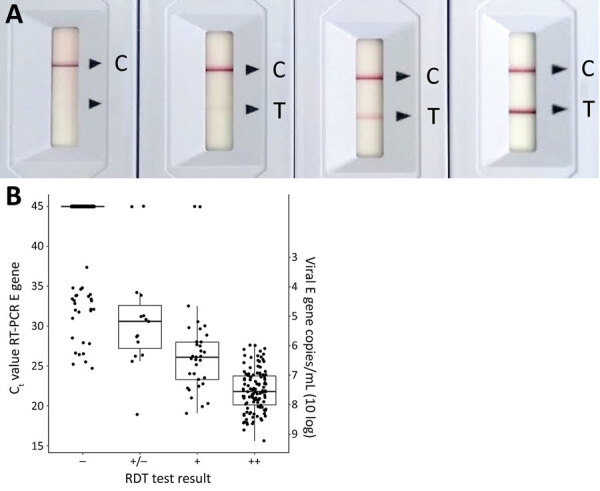
Comparison of results for rapid antigen detection tests and PCR for diagnosis of severe acute respiratory syndrome coronavirus 2, the Netherlands. A) Example of the 4 grade scaling system used for result readout. Results were determined by the absence or presence of the T band as well as band intensity. An absent T band is read as negative. Positive results were further distinguished as follows: very faint band, +/−; medium intensity band, +; and strong band, ++. Final readout of results was done after the manufacturer’s recommended 15 minutes. B) Correlation of RT-PCR C_t_ and Ag RDT test band intensity. RT-PCR C_t_ results were grouped by the 4 categories of the Ag RDT result readout (n = 970). Horizontal line in each box indicates median C_t_; box borders indicate 75% interquartile range (IQR), whiskers represent the range of values 1.5 times the IQR, and dots represent individual test results. Ag RDT, antigen rapid detection test; C, control; C_t_, cycle threshold; E gene, envelope gene; RT-PCR, reverse transcription pPCR; T, test; −, negative; +/− weak positive; +, positive; ++, strong positive.

For the Ag RDT, the SD Biosensor SARS-CoV-2 rapid antigen test distributed by Roche (reference no. 9901-NCOV-01G; lot no. QCO3020079/Sub:A-2) was performed immediately onsite following manufacturer’s instructions. A 4-grade scaling readout (−; +/−, +; ++) representing the strength of the test band was used (Figure 1, panel A). Time until positive results was logged as <5 min, <10 min (not part of the manufacturer’s instructions for use), or 15 min; recommended readout was 15–30 min. When results were dubious (i.e., test line barely visible or labeled as +/− but regarded as positive test result), 2 persons performed the readout.

### Data Analysis

We merged data from the Ag RDT, RT-PCR, virus culture, and clinical questionnaire using Microsoft Access and data performed analysis using R version 4.0.2 (The R Project for Statistical Computing, https://www.r-project.org). Sensitivity and specificity of Ag RDT were calculated in relation to the RT-PCR results. Wilcoxon score interval was used to determine CIs of proportions.

## Results

### Characteristics of Study Population

During the study period of October 9–15, 2020, a total of 970 (26.8%) of 3,615 persons visiting the testing site were included in the study; inclusion was put on hold occasionally during the day when testing posts became crowded. The average age of study participants was 42 years (range 18–86 years); most were female (n = 525, 54.7%). Among the participants manifesting symptoms, 73.4% had symptom onset <7 days (n = 650/886). Most (84.9%) of the samples had high viral load (PCR C_t_ <30, envelope gene (E gene) 2.17 × 10^5^ copies/mL) (Table 1). The age and sex distribution of study participants was representative of the tested population in general: average age 38.4 years, 57% female (data not shown). We did not record reasons for not participating.

**Table 1:** Characteristics of the population of study comparing rapid antigen test and PCR for severe acute respiratory syndrome coronavirus 2, the Netherlands*

Characteristic	Value
Total	970
Median age, y (range)	42 (18–86)
Sex	
M	435 (44.8)
F	525 (54.1)
Unknown	10 (1.1)
Symptoms reported	886 (91.3)
Days after symptom onset, median (no. cases/total no. tested)	4 (725/970)
0–3	319 (44.0)
4–7	331 (45.7)
>8	75 (10.3)
Positivity by PCR	186 (19.2)
PCR C_t_ E gene, median (range)	23.6 (15.6–37.4)
C_t_ >35	1 (0.5)
C_t_ >30	28 (15.1)
C_t_ <30	159 (85.5)
C_t_ <25	113 (60.8)
C_t_ <20	31 (16.7)

*Values are no. (%) except as indicated. E gene, envelope gene; C_t_, cycle threshold.

At the time of requesting the appointment, most participants (91.3%) had symptoms; most frequently reported were common cold symptoms, such as runny nose (64.5%), sore throat (57%), coughing (55%), headache (48%), tiredness (38%), muscle pain (27%), shortness of breath (21%), and chills (21%). Some of the more typical and serious symptoms such as fever and reproductive cough were reported by 17% of participants. A very small percentage (1.5%) reported loss of taste and smell.

### Performance of the Ag RDT

The overall sensitivity of the Ag RDT was 84.9% (95% CI 79.1%–89.4%) (Tables 2, 3). Positive predictive value was 97.5% (95% CI 93.8%–99.0%) under an average of 19.2% current prevalence in the region calculated by PCR positivity rate. Sensitivity improved considerably when analyzed by various PCR C_t_ intervals showing highest sensitivity for C_t_ <25 (4.87 × 10^6^ E gene copies/mL); sensitivity was 99.1% (95% CI 95.2%–100%). For C_t_ <30 (2.17 × 10^5^ E gene copies/mL), sensitivity was 94.3% (95% CI 89.6%–97.0%). Sensitivity among participants that sought care within 3 days after disease onset was higher (94.9%) than for participants who came later in their disease progression (90.6%) (Table 3). Hence, sensitivity was strongly associated with viral load. PCR-positive samples that were not positive by Ag RDT (n = 28) showed a mixed distribution of viral load (C_t_ <30 for 10/28 samples). Date of onset was available for 16/28 patients; 12/28 tested <7 days after onset. Of the 28 samples, 5 were cultivable (2 samples were not cultured); all 5 had C_t_ <30 and onset <7 days. Only 2/28 had no symptoms but had contact with a confirmed case (average C*_t_* 33).

**Table 2:** Overview of results of comparison of rapid antigen test and PCR for severe acute respiratory syndrome coronavirus 2, the Netherlands*

Ag RDT result	PCR result		Total
	Positive	Negative	
Positive	158	4	162
Negative	28	780	808
Total	186	784	970

*Ag RDT, rapid antigen detection test.

**Table 3:** Characteristics of rapid antigen detection test compared with reverse transcription PCR stratified by days after symptom onset, the Netherlands*

	0–3 d past onset			0–7 d past onset			All		
Characteristic	No.	% (95% CI)		No.	% (95% CI)		No.	% (95% CI)	
Clinical sensitivity	319	94.9 (86.1–98.3)		650	90.6 (84.3–94.6)		970	84.9 (79.1–89.4)	
Sensitivity C*_t_* <30	316	98.2 (90.6–99.9)		640	95.8 (90.5–98.2)		943	94.3 (89.6–0.97)	
Sensitivity C_t_ <25	305	100 (92.1–100)		608	98.8 (93.7–99.9)		897	99.1 (95.2–100)	
Clinical specificity	319	99.6 (97.9-100)		650	99.6 (98.6-99.9)		970	99.5 (98.7-99.8)	
Positive predictive value	NA	98.2 (90.7–99.9)		NA	98.3 (94.0–99.5)		NA	97.5 (93.8–99.0)	
Negative predictive value	NA	98.9 (96.7–99.6)		NA	97.7 (96.1–98.7)		NA	96.5 (95.0–97.6)	

*Sensitivity and specificity of Ag RDT was calculated based on reverse transcription PCR results and days since symptoms onset. Positive and negative predictive values were calculated using 19.2% prevalence setting. Ag RDT, rapid antigen detection test; C_t_, cycle threshold; NA, not applicable.

The overall specificity of Ag RDT was 99.5% (95% CI 98.7%–99.8%); negative predictive value was 96.5% (95% CI 95.0%–97.6%), which increased with shorter time after symptom onset (Table 3). Three of 4 samples negative by PCR (and culture) that were positive by Ag RDT were negative by RT-PCR for other respiratory viruses; 1 was weakly positive for rhinovirus (C_t_ >35). Metagenomic sequencing confirmed rhinovirus B.

### Association of Ag RDT Results with Infectivity

A total of 176/186 specimens that tested positive by Ag RDT, RT-PCR, or both were inoculated on Vero cells; 140 (79.5%) were culture positive after 7 days of cell culture. We observed cytopathic effect 2–5 days after inoculation. The culture-positive specimens were obtained from persons at a median of 4 days post onset of disease (range 1–12 days) and high viral load (average C_t_ 22.8, viral load 6.99 × 10^7^ E gene copies/mL). Median days past symptom onset did not differ between Ag RDT and PCR positive samples independently of successful culture (Table 5).

**Table 5:** Comparison of rapid test, PCR, and culture results for severe acute respiratory syndrome coronavirus 2, the Netherlands*

RDT result	Culture result	PCR C_t_											Total
		<20			20–25			25–30			>30		
		No.	Median days after onset (range)†		No.	Median days after onset (range)‡		No.	Median days after onset (range)§		No.	Median days after onset (range)¶	
+	NA	1	2		4	3.5		4	6.5		0	NA	9
+	+	30	3		74	4		30	4		1	9	135
+	−	0	NA		3	5		4	5.5		7	7	14
−	NA	0	NA		0	NA		1	3		0	NA	1
−	+	0	NA		1	7		4	5.5		0	NA	5
−	−	0	NA		0	NA		3	NA		19	6.5	22
Total		31	3 (1–9)		82	4 (1–12)		46	4 (1–9)		27	7 (2–15)	186

*No. indicates no. participants. C_t_, cycle threshold; NA, not applicable. †Unknown for 9 participants.  ‡Unknown for 16 participants. §Unknown for 12 participants. ¶Unknown for 9 participants.

**Figure 2:**
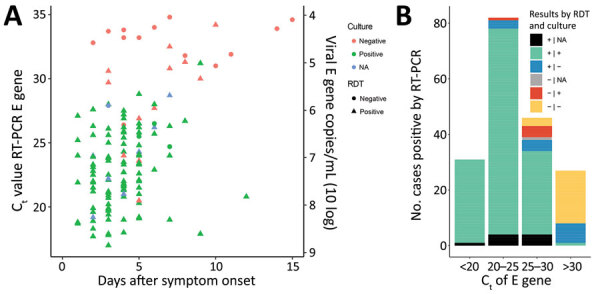
Relationships of time from symptom onset to testing and cycle threshold values to results for rapid antigen detection tests and PCR for diagnosis of severe acute respiratory syndrome coronavirus 2, the Netherlands. A) Cycle thresholds of positive samples in relation to days since symptom onset, Ag RDT positivity, and culture outcomes of participation with known disease onset date (n = 140). B) PCR-positive samples by cycle threshold (n = 186) in relation to Ag RDT and culture test results. Ag RDT, antigen rapid detection test; C_t_, cycle threshold; E gene, envelope gene; NA, not available; RT-PCR, reverse transcription PCR.

Of the 140 cultured specimens, 5 (3.6%) were Ag RDT negative. These specimens were collected a median of 6 days after onset of disease (range 5–7 days; 2 values missing) and had high viral loads (average C_t_ 25.7, viral load 3.15 × 10^6^ E gene copies/mL). In samples with C_t_ <30 (<2.17 × 10^5^ E gene copies/mL), 10/176 (6%) could not be cultured and 4/176 (2%) were not detectable by Ag RDT. For samples with C_t_ >30, 1/27 (4%) could be cultured and 8/27 (30%) were Ag RDT positive. These data indicate that for C_t_ >30 (2.17 × 10^5^ E gene copies/mL), most samples are not cultivable, which is in agreement with previously published data (*8*,*9*) (Table 5; Figure 2).

### Significance of Time to Result

We logged results at 3 time points: 5 minutes, 10 minutes, and the recommended readout time of 15 minutes; we recorded intensity of the test band. In general, most (95%) strong positive samples appeared <5 min after sample addition. Test bands showing medium intensity had a more equal distribution of time to results in the 3 timeframes, whereas most (73%) weak positive bands required the recommended 15-minute readout (Table 4). Band intensity correlated with viral load (Figure 1).

**Table 4:** Results of rapid antigen detection test for severe acute respiratory syndrome coronavirus 2, the Netherlands*

Time to result	Result, no. (%)				Total
	−	+/−	+	++	
5 min	NA	1 (7)	8 (24)	108 (95)	117
10 min	NA	3 (20)	12 (36)	4 (4)	19
15 min	NA	11 (73)	13 (39)	2 (1)	26
Total tests	808	15	33	114	970

*Results of the Ag RDT were recorded at 3 time points: 5 min, 10 min, and the manufacturer-recommended 15 min. If result between first and last readout did not change, the first was registered as final result. Ag RDT, rapid antigen detection test; NA, not applicable; −, negative; +/− weak positive; +, positive; ++, strong positive.

## Discussion

We describe the results of a large clinical evaluation study using an antigen rapid test in a medium-high prevalence setting in a symptomatic, nonhospitalized population to detect SARS-CoV-2 infections. Overall, the test performed well, detecting 84.9% of all cases with RT-PCR as reference. Our results align well with data from other independent evaluations, including low rate of false positivity (*4*). A question to address is if and how Ag RDT can identify infectious persons and support the test, trace, and isolate strategy employed worldwide to control the COVID-19 pandemic. In our evaluation, we have identified ≈97% of persons with sufficient viral load to enable virus culture; this finding suggests that Ag RDT alone in this population would have a high sensitivity for identifying infectious persons. On the basis of its performance in our study, the test would fulfill World Health Organization criteria until the prevalence of SARS-CoV-2 drops below 2.5% based on positive predictive value.

One of the unique strengths of this study is the correlation of results with infectivity. Most PCR positive samples with high viral load could be cultured successfully; however, a fraction of a potentially infectious group was not detected by the Ag RDT. These patients were generally tested in the later phase of the infection but still had a high viral load and positive virus cultures. Although the presence of antibodies in patients after the first week of onset could reduce the sensitivity of Ag RDT, this possibility does not explain the discrepancy in the samples that were negative by the RDT and positive by virus culture; we previously demonstrated that the presence of neutralizing antibodies does inversely correlate with virus culture (*9*). One possible explanation is the use of different swabs, causing discrepancy in viral load in the RT-PCR and culture versus Ag RDT samples. However small the proportion, missing infectious persons can have serious consequences in specific populations. Testing algorithms should therefore be carefully aligned to high-risk and high-priority groups. On the other hand, Ag RDT could detect cases with relatively low viral load with high sensitivity, thereby providing a safety margin around the suggested threshold of infectiousness.

In asymptomatic persons, the absence of symptoms might make them less cautious, whereby they do contribute to the spread of the virus. Previous reports have shown that asymptomatic persons have similar viral loads to symptomatic persons (*9*,*10*); therefore, the Ag RDT could be used in this population. Because performance data of Ag RDT in this specific population is scarce as of March 2021, additional validation of the Ag RDT test is recommended. Repeated testing following the calculated incubation time will provide more test certainty.

Several Ag RDTs are on the market; most use nasopharyngeal swabs for sampling. Oropharyngeal and nasopharyngeal swabs are considered the best sample types for detecting SARS-CoV-2 especially in the early phase (*2*,*10*). However, the swabbing requires trained personnel and causes discomfort to the patient. Only a few Ag RDTs are marketed directly with a less invasive sample, the nasal swab. The available performance data indicates no notable difference between Ag RDT and RT-PCR in detecting symptomatic cases, and the use of the more superficially collected nasal swab specimens seems to be a good alternative (N. Van der Moeren et al., unpub. data, https://doi.org/10.1101/2020.10.19.20215202). Investigators can further explore the use of self-sampling, which is one of the potential directions Ag RDT testing will take because it does not require trained personnel, reduces infection risk for the healthcare worker who takes the swab sample, and enables testing for a wider population. Studies indicate that self-sampling is somewhat less precise than sampling by trained professionals, further lowering detection rate (*11*); evaluation studies are ongoing.

One limitation of our study is that, in our setting, we compared results of RT-PCR and Ag RDT; however, in contrast to the instructions for 1 swab specimen for the Ag RDT, 2 swab specimens were taken for RT-PCR and virus culture, which probably resulted in a higher amount of viral material collected. This difference might explain some of the discrepancies between Ag RDT and PCR or culture. Furthermore, the same nostril was used to take the second swab for the Ag RDT, which was meant to grant comparability between the 2 tests but might have resulted in lower viral load in the second sample. We used culture as a correlate of infectivity, which has certain limitations but is the best available technique to measure infectivity. Recall bias by the study enrollees when filling out the questionnaires could have affected the data provided. Furthermore, testing is free of charge only for persons who had either relevant symptoms or notified contact with an infected person; therefore, some persons might have provided symptoms falsely to be tested for other reasons.

We conclude that the use of Ag RDT in our drive-through test stations would provide a good method to identify most infectious patients. The logistics of implementation crucial for further rollout include a safe working environment for personnel performing the assays if implemented onsite and a system that enables follow-up testing by PCR for risk groups. The national outbreak management team of the Netherlands recommends using Ag RDT for rapid screening but cautions against sole use of Ag RDTs in vulnerable persons, such as those at risk for severe illness and those living or working in long-term care facilities, because of the potential of false negative cases. Whereas a positive Ag RDT can be used to trigger contact tracing and isolation, it is imperative to inform patients about the potential for false negative testing, and the need for continued behavioral measures. A slightly higher risk for missed cases is debatable in patients who have little contact with high-risk persons, although the identification of these cases will be challenging. Ideally, rapid antigen testing should be secured through a triage system that guides patients to the proper testing algorithm and includes repeated testing.
